# Sleep disorders associated with risk of prostate cancer: a population-based cohort study

**DOI:** 10.1186/s12885-019-5361-6

**Published:** 2019-02-13

**Authors:** Wei-Sheng Chung, Cheng-Li Lin

**Affiliations:** 1 Department of Internal Medicine, Taichung Hospital, Ministry of Health and Welfare, No. 199, Section 1, San-Min Road, West District, Taichung City, 40343 Taiwan; 2 Department of Health Services Administration, China Medical University, Taichung, Taiwan; 3 Department of Healthcare Administration, Central Taiwan University of Science and Technology, Taichung, Taiwan; 4 Management Office for Health Data, China Medical University Hospital, Taichung, Taiwan; 5 College of Medicine, China Medical University, Taichung, Taiwan

**Keywords:** Sleep disorders (SDs), Prostate cancer, Cohort study

## Abstract

**Background:**

Disrupted sleep rhythms may lead to cancer development. We conducted a population-based cohort study to evaluate the incidence and risk of prostate cancer in patients with sleep disorders (SDs).

**Methods:**

Patients newly diagnosed with SDs between 2000 and 2010 were enrolled from the Taiwan Longitudinal Health Insurance Database. A non-SD cohort age-matched (5-y intervals), comorbidities, and medications was randomly sampled from the general population at a 1:1 ratio. The follow-up period extended from the index date of SDs to the diagnosis of prostate cancer, censoring, or the end of 2013. We used Cox proportional hazards models to calculate the risk of prostate cancer.

**Results:**

In total, 41,444 patients were enrolled in each cohort. The mean age of the SD cohort was 48.0 years and that of the non-SD cohort was 47.8 years, with 58.2% of both cohorts aged younger than 50 years. The incidence of prostate cancer increased with age. The overall incidence of prostate cancer was higher in the SD cohort than in the non-SD cohort (9.56 vs 6.36 per 10,000 person-y), with an adjusted hazard ratio of 1.42 (95% CI = 1.20–1.69). Age-specific analysis revealed a 1.35-fold increased risk of prostate cancer in the patients aged ≥65 years in the SD cohort compared with the non-SD counterparts (95% CI = 1.10–1.65).

**Conclusions:**

Patients with SDs are associated with increased risk of prostate cancer.

## Background

Sleep disorders (SDs) are one of the most common problems in the general population. The cause of SDs can be a primary disorder or secondary to various psychiatric and medical illnesses. The prevalence of SDs tends to increase with age. Approximately 41% of the elderly experienced difficulty initiating sleep onset insomnia, sleep maintaining insomnia, or early morning awakening insomnia. [1] Inadequate and nonrestorative sleep impairs quality of life and lead to future depression development and adverse health consequences. [2–5]

Previous studies have suggested that sleep disruption and circadian dysrhythmia may increase the risk of breast cancer in women. [6, 7] Melatonin, a pineal hormone, is related to circadian rhythm and sleep. [8] Recent studies have indicated that melatonin carries potentially chemopreventive, oncostatic, and anticarcinogenic effects. [9, 10] Prostate cancer has become a major public health issue in men worldwide, though the etiology of the disease remains elucidative. Two studies have reported that short sleep duration is associated with increased risk of prostate cancer. [11, 12] However, Markt et al. [13] conducted a prospective study and did not find association between sleep duration and risk of prostate cancer.

Prostate cancer is a leading cancer in men and causes a considerable economic and public health burden. [14] The incidence of prostate cancer has rapidly increased from 26.2 per 100,000 population in 2002 to 47.9 per 100,000 population in 2012 in Taiwan. [15] Employment with high job strain and stress might contribute to excess risk of prostate cancer. [16] The residents in urban areas may be associated with prostate cancer. [17] Adult obesity in the epidemiologic study showed association with development of prostate cancer. [18] Urinary stones may lead to obstruction, infection, and further cancer development. [19] A nested case-control study indicated an association between SDs and cancer. [20] People use hypnotics, mainly benzodiazepines and nonbenzodiazepine agents, to aid sleep or treat anxiety. However, hypnotic use may be related to increased cancer risk. [21] Therefore, we conducted a large population-based cohort study to investigate the risk of prostate cancer in patients with SDs compared with people without SDs after controlling for hypnotic use and potential covariates.

## Methods

### Data source

We conducted a retrospective population-based cohort study by using the Taiwan Longitudinal Health Insurance Database (LHID). The Taiwan government launched the National Health Insurance (NHI) program in 1995. Approximately 99% of the total population of approximately 23 million people, participate in the program. [22] The LHID is a sub-database of the National Health Insurance Research Database (NHIRD), which was established by the National Health Insurance Administration (NHIA) and is maintained by the National Health Research Institutes. The LHID contains the longitudinally linked data of 1,000,000 enrollees randomly sampled from the NHIRD. The LHID was released with de-identified data, rendering researchers unable to identify the study patients. Diseases in the database are coded according to the 2001 International Classification of Diseases, Ninth Revision, Clinical Modification (ICD-9-CM). The institutional review board (IRB) of China Medical University Hospital approved this study (IRB ID number: CMUH104-REC2–115).

### Sampled patients

To evaluate the risk of prostate cancer in patients with SDs, we compared a SD cohort with a non-SD cohort. From the LHID, we selected male patients who received a first diagnosis of SDs (ICD-9-CM codes 307.4 including nonorganic sleep disorders, insomnia, and circadian rhythm SD, 327 organic sleep disorders, and 780.5 indicating sleep disturbance) between January 1, 2000 and December 31, 2010, and set the first diagnosis day of SDs as the index date. We assembled the non-SD cohort by randomly selecting male patients without a diagnosis of SDs from the LHID, and frequency-matched them with the SD cohort patients by age (5-y intervals), occupation, urbanization level, comorbidities, and medications at a 1:1 ratio. We set the index date of the matched cases as the index date for the non-SD patients. We enrolled only patients who were aged more than 20 years and who did not have a history of prostate cancer (ICD-9-CM code 185) before the index date.

### Outcomes, occupation, urbanization level, comorbidities, and medication

All patients were followed until a diagnosis of prostate cancer, withdrawal from the NHI, death, or the end of 2011. We categorized the occupation variable into white collar (working with long indoor work hours, such as business and administration personnel), blue collar (working with long outdoor work hours, such as farmers and laborers), and others (primarily retired, unemployed, and low-income groups). The urbanization variable for the patients’ residing area was categorized into four levels: level 1 being the highest urbanization and level 4 being the least urbanization. We examined pre-existing comorbidities including hyperlipidemia (ICD-9-CM code 272), diabetes (ICD-9-CM code 250), hypertension (ICD-9-CM codes 401–405), urinary stones (ICD-9-CM codes 592.0. 592.1, 594.0, and 594.1), urinary tract infection (ICD-9-CM codes 590 and 595), obesity (ICD-9-CM code 278), anxiety (ICD-9-CM code 300.00), depression (ICD-9-CM codes 296.2, 296.3, 300.4, and 311), chronic obstructive pulmonary disease (COPD, ICD-9-CM codes 491, 492, 496), and alcohol-related illness(ICD-9-CM codes 291, 303, 305, 571.0, 571.1, 571.2, 571.3, 790.3, and V11.3). A medication history of hypnotics and antihypertensive medication use was included in the analysis. In addition, we also evaluated prostate specific antigen (PSA) screening in the study.

### Statistical analysis

The demographic and clinical characteristics of the SD and non-SD cohorts, including age (≤ 49, 50–64, and ≥ 65y), occupation category (white collar, blue collar, and others), urbanization level, comorbidities, and medication treatments, were compared using the chi-squared test. For continuous variables, we conducted the Student *t* test to compare the SD and non-SD cohorts. We computed the incidence rate (per 10,000 person-y) of follow-up for each cohort. To evaluate the risk of prostate cancer for the SD cohort compared with the non-SD cohort, hazard ratios (HRs) and 95% confidence intervals (CIs) were estimated using univariable and multivariable Cox proportional hazards models. The multivariable models were simultaneously adjusted for age; occupation category; urbanization level; comorbidities of hyperlipidemia, diabetes, hypertension, urinary stones, urinary tract infection, obesity, anxiety, and depression; and medication of hypnotics as well as antihypertensive medication, and PSA screening. The cumulative incidence of prostate cancer was calculated using the Kaplan–Meier method and the difference was evaluated using the log-rank test. All analyses were conducted using SAS statistical software (Version 9.4 for Windows; SAS Institute, Inc., Cary, NC, USA), with statistical significance set at *P* < .05 for a 2-tailed test.

## Results

The SD and non-SD cohorts each comprised 41,444 patients. The mean age of the SD cohort was 48.0 years and that of the non-SD cohort was 47.8 years, with 58.2% of both cohorts aged less than 50 years (Table 1). Most of the patients in both cohorts had white-collar jobs (54.3% vs 54.0%) and tended to reside in an urbanized area (59.6% vs 59.1%). The proportions of urinary tract infection and PSA screening in the SD cohort were significantly higher than those in the non-SD cohort.

**Table 1:** Comparison of Demographics and Comorbidities of Patients With and Without SDs

	Sleep disorder (*N* = 41,444)		Control (*N* = 41,444)		*P* value
	*n*	%	*n*	%	
Age, year					0.58
< 49	24,137	58.2	24,114	58.2	
50–64	9629	23.2	9597	23.2	
≥ 65	7701	18.6	7710	18.6	
Mean (SD) ^#^	48.0	16.6	47.8	16.7	034
Occupation					0.65
White collar	22,483	54.3	22,367	54.0	
Blue collar	14,250	34.4	14,377	34.7	
Others^‡^	4711	11.4	4700	11.3	
Urbanization level^†^					0.22
1 (highest)	12,282	29.6	12,015	29.0	
2	12,417	30.0	12,472	30.1	
3	7350	17.7	7436	17.9	
4 (lowest)	9395	22.7	9521	23.0	
Comorbidity					
Hyperlipidemia	7524	18.2	7630	18.4	0.34
Diabetes	2187	5.28	2238	5.40	0.43
Hypertension	12,345	29.8	12,459	30.1	0.39
Urinary stones	2770	6.68	2868	6.92	0.18
Urinary tract infection	3039	7.33	2796	6.75	0.001
Obesity	312	0.75	338	0.82	0.31
Anxiety	1775	4.28	1691	4.08	0.14
Depression	1187	2.86	1191	2.87	0.93
COPD	4939	11.9	5033	12.1	0.32
Alcohol-related illness	1573	3.80	1566	3.78	0.90
Medication					
Hypnotics	23,308	56.2	23,082	55.7	0.11
Antihypertensives	11,657	28.1	11,748	28.4	0.48
PSA screening	5729	13.8	4523	10.9	< 0.001

Chi-square test compared to total SD; ^#^:*t* test; COPD: Chronic obstructive pulmonary disease

^†^: The urbanization level was categorized into 4 levels according to the population density of the residential area, with level 1 indicating the highest urbanization and level 4 indicating the lowest urbanization

**Fig. 1:**
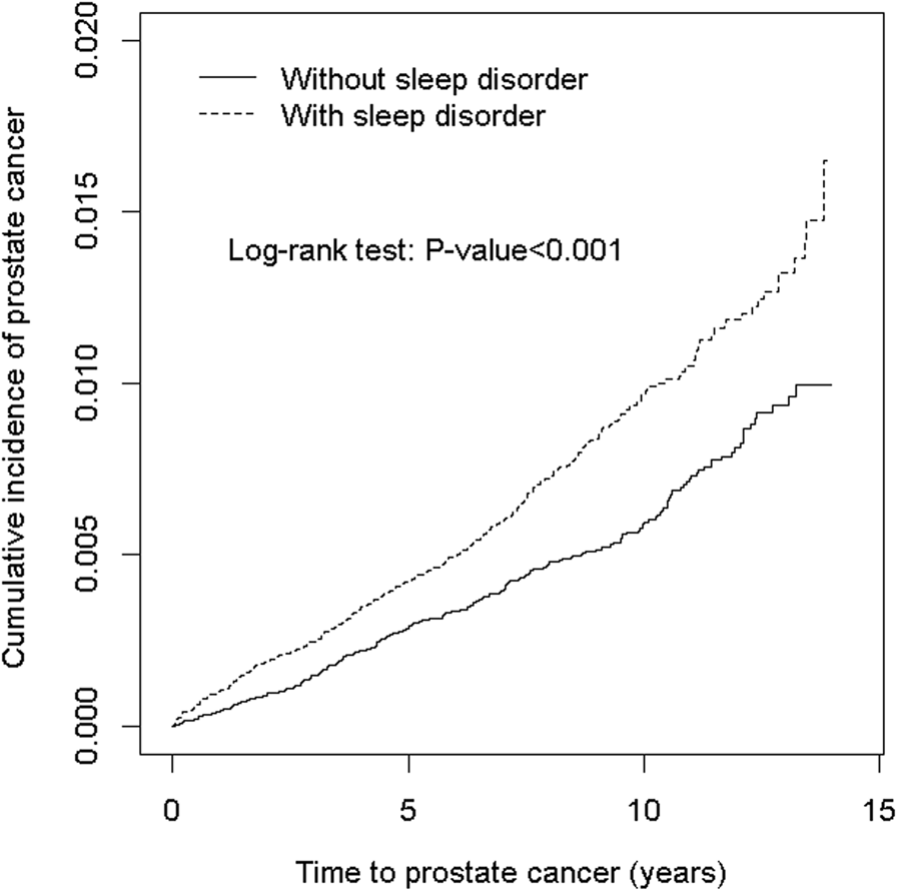
Comparison of Kaplan-Meir analysis-determined cumulative incidence of Prostate cancer of SD and non-SD cohorts

The overall incidence of prostate cancer was 51% greater in the SD cohort than in the non-SD cohort (9.56 vs 6.36 per 10,000 person-years), with an adjusted HR (aHR) of 1.42 (95% CI = 1.20–1.69) (Table 2). The cumulative incidence of prostate cancer was greater in the SD cohort than in the non-SD cohort (Fig. 1). Age-specific analysis revealed a significantly higher risk of developing prostate cancer in the patients all aged group in the SD cohort compared with the same age group in the non-SD cohort. Occupation category-specific analyses showed that among the patients employed in white-collar positions, those with SDs had a significantly higher risk of prostate cancer than did those without SDs (aHR = 1.67, 95% CI = 1.28–2.18). The SD cohort again exhibited a significantly higher risk of prostate cancer compared with the non-SD cohort when only the patients living in the 2nd highest (aHR = 1.43, 95% CI = 1.03–1.98), 3rd highest (aHR = 1.79, 95% CI = 1.11–2.91), and lowest (aHR = 1.42, 95% CI = 1.03–1.95 for lowest) urbanization level areas were considered. In patients without comorbidities, the risk of prostate cancer was 2.26-fold higher in the SD cohort than in the non-SD cohort (95% CI = 1.46–3.51). Among the patients not prescribed the examined medications, those with SDs had a higher risk of prostate cancer than did those without SDs (aHR = 1.46, 95% CI = 1.07–1.99 for those not prescribed hypnotics; aHR = 1.66, 95% CI = 1.26–2.19 for those not prescribed antihypertensive medication). In patients without PSA screening, the risk of prostate cancer was 1.58-fold higher in the SD cohort than in the non-SD cohort (95% CI = 1.26–1.98).

**Table 2:** Comparison of Incidence Densities of Prostate Cancer Hazard Ratios of Men With and Without SDs Stratified by Demographic Characteristics and Comorbidities

	Sleep disorder							
	Yes			No				
	Event	PY	Rate^#^	Event	PY	Rate^#^	Crude HR (95% CI)	Adjusted HR^†^ (95% CI)
All	327	342,189	9.56	222	349,060	6.36	1.51(1.28, 1.80)***	1.42(1.20, 1.69)***
Age								
< 49	14	208,908	0.67	4	214,073	0.19	3.82(1.26, 11.6)*	3.12(1.02, 9.58)*
50–64	94	79,239	11.9	58	81,376	7.13	1.70(1.23, 2.36)**	1.50(1.08, 2.08)*
≥ 65	219	54,042	40.5	160	53,610	29.8	1.36(1.11, 1.67)**	1.35(1.10, 1.65)**
P for trend								0.23
Occupation								
White collar	146	187,392	7.79	89	193,265	4.61	1.71(1.31, 2.23)***	1.67(1.28, 2.18)***
Blue collar	118	118,498	9.96	96	118,302	8.11	1.23(0.94, 1.61)	1.14(0.87, 1.49)
Others^‡^	63	36,300	17.4	37	37,493	9.87	1.77(1.18, 2.66)**	1.56(1.04, 2.36)*
P for trend								0.06
Urbanization level								
1 (highest)	97	99,276	9.77	74	104,986	7.05	1.40(1.04, 1.90)*	1.30(0.96, 1.77)
2	88	103,147	8.53	60	104,878	5.72	1.51(1.08, 2.09)*	1.43(1.03, 1.98)*
3	46	61,671	7.46	26	61,550	4.22	1.78(1.10, 2.88)*	1.79(1.11, 2.91)*
4 (lowest)	96	78,094	12.3	62	77,646	7.98	1.55(1.12, 2.13)**	1.42(1.03, 1.95)*
P for trend								0.49
Comorbidity^‡^								
No	66	171,700	3.84	29	178,753	1.62	2.43(1.57, 3.76)***	2.26(1.46, 3.51)***
Yes	261	170,489	15.3	193	170,307	11.3	1.36(1.13, 1.64)**	1.30(1.08, 1.56)**
P for trend								0.02
Hyperlipidemia								
No	221	281,496	7.85	141	288,143	4.89	1.62(1.31, 2.00)***	1.52(1.23, 1.88)***
Yes	106	60,693	17.5	81	60,918	13.3	1.32(0.99, 1.76)	1.24(0.93, 1.66)
P for trend								0.27
Diabetes								
No	288	326,577	8.82	188	333,506	5.64	1.58(1.31, 1.90)***	1.47(1.22, 1.77)***
Yes	39	15,611	25.0	34	15,555	21.9	1.14(0.72, 1.81)	1.15(0.72, 1.82)
P for trend								0.21
Hypertension								
No	132	245,636	5.37	70	253,208	2.76	1.99(1.49, 2.65)***	1.79(1.34, 2.40)***
Yes	195	96,553	20.2	152	95,853	15.9	1.27(1.03, 1.58)*	1.24(1.00, 1.54)*
P for trend								0.58
Urinary stones								
No	302	319,449	9.45	203	326,940	6.21	1.54(1.29, 1.83)***	1.45(1.21, 1.73)***
Yes	25	22,740	11.0	19	22,120	8.59	1.29(0.71, 2.34)	1.26(0.69, 2.31)
P for trend								0.60
Urinary tract infection								
No	290	321,203	9.03	197	330,283	5.96	1.53(1.27, 1.83)***	1.44(1.20, 1.73)***
Yes	37	20,986	17.6	25	18,777	13.3	1.33(0.80, 2.20)	1.32(0.79, 2.20)
P for trend								0.60
Obesity								
No	222	346,734	6.40	323	339,663	9.51	1.50(1.26, 1.78)***	1.40(1.18, 1.66)***
Yes	0	2326	0.00	4	2525	15.8	–	–
P for trend								0.95
Anxiety								
No	310	329,242	9.42	217	335,558	6.47	1.47(1.23, 1.75)***	1.39(1.17, 1.65)***
Yes	17	12,947	13.1	5	13,502	3.70	3.53(1.30, 9.58)*	2.72(0.99, 7.48)
P for trend								0.09
Depression								
No	317	332,890	9.52	216	339,164	6.37	1.51(1.27, 1.79)***	1.43(1.20, 1.70)***
Yes	10	9299	10.8	6	9897	6.06	1.82(0.66, 5.00)	1.15(0.41, 3.28)
P for trend								0.74
COPD								
No	233	304,824	7.64	156	313,361	4.98	1.55(1.27, 1.90)***	1.44(1.17, 1.76)***
Yes	94	37,365	25.2	66	35,699	18.5	1.36(0.99, 1.86)	1.35(0.99, 1.85)
P for trend								0.50
Alcohol-related illness								
No	318	331,360	9.60	217	338,475	6.41	1.51(1.27, 1.79)***	1.41(1.19, 1.68)***
Yes	9	10,829	8.31	5	10,585	4.72	1.74(0.58, 5.20)	2.72(0.81, 9.11)
P for trend								0.80
Medication								
Hypnotics								
No	97	143,651	6.75	68	142,627	4.77	1.42(1.04, 1.93)*	1.46(1.07, 1.99)*
Yes	230	198,538	11.6	154	206,434	7.46	1.57(1.28, 1.93)***	1.42(1.15, 1.74)***
P for trend								0.59
Antihypertensives								
No	140	252,468	5.55	82	260,095	3.15	1.79(1.36, 2.35)***	1.66(1.26, 2.19)***
Yes	187	89,721	20.8	140	88,966	15.7	1.33(1.07, 1.65)*	1.28(1.02, 1.59)*
P for trend								0.11
PSA screening								
No	193	289,171	6.67	126	305,769	4.12	1.60(1.28, 2.01)***	1.58(1.26, 1.98)***
Yes	134	53,018	25.3	96	43,292	22.2	1.18(0.91, 1.54)	1.20(0.92, 1.56)
P for trend								0.047

Rate^#^, incidence rate per 10,000 person-years; Crude HR, relative hazard ratio

Adjusted HR^†^: multivariable analysis with adjustment for age; occupation category; urbanization level; comorbidities of hyperlipidemia, diabetes, hypertension, urinary stones, urinary tract infection, obesity, anxiety, depression, chronic obstructive pulmonary disease, and alcohol-related illness, and medication of hypnotics as well as antihypertensive medication, and PSA screening; Comorbidity^‡^: Only one comorbidity (including hyperlipidemia, diabetes, hypertension, urinary stones, urinary tract infection, obesity, anxiety, and depression) classified as the comorbidity group

*P* < .05, **P < .01, ***P < .001

The analysis of HRs for developing prostate cancer was stratified by follow-up time. The SD cohort exhibited a significantly increased risk of prostate cancer compared with the non-SD cohort in follow-up time of ≤1 year (aHR = 2.46, 95% CI = 1.40–4.33) and > 5 years (aHR = 1.36, 95% CI = 1.07–1.73) (Table 3).

**Table 3:** Trends of Prostate Cancer Risk Stratified by Follow-Up Years

	Sleep disorder							
	Yes			No				
Follow-up time, years	Event	PY	Rate^#^	Event	PY	Rate^#^	Crude HR (95% CI)	Adjusted HR^†^
								(95% CI)
≤1	42	41,067	10.2	17	41,066	4.14	2.47(1.41, 4.34)**	2.46(1.40, 4.33)**
2–3	58	79,920	7.26	42	79,951	5.25	1.38(0.93, 2.06)	1.37(0.92, 2.03)
4–5	65	72,438	8.97	52	72,465	7.18	1.25(0.87, 1.80)	1.20(0.83, 1.73)
> 5	162	148,765	10.9	111	155,578	7.13	1.54(1.21, 1.96)***	1.36(1.07, 1.73)***

Rate^#^, incidence rate per 10,000 person-years; Crude HR, relative hazard ratio

Adjusted HR^†^: multivariable analysis with adjustment for age; occupation category; urbanization level; comorbidities of hyperlipidemia, diabetes, hypertension, urinary stones, urinary tract infection, obesity, anxiety, depression, chronic obstructive pulmonary disease, and alcohol-related illness,; and medication of hypnotics as well as antihypertensive medication, and PSA screening

*P < .05, **P < .01

The risk of developing prostate cancer increased with age (aHR = 1.10, 95% CI = 1.09–1.11 every 1 y). Compared to patient of others occupation, patients of white collar occupation had a higher risk of developing prostate cancer (aHR = 1.36, 95% CI = 1.06–1.74). The risk of developing prostate cancer was greater for patients with comorbidities of hyperlipidemia (aHR = 1.36, 95% CI = 1.13–1.64), diabetes (aHR = 1.36, 95% CI = 1.06–1.76), and PSA screening (aHR = 1.99, 95% CI = 1.67–2.36) (Table 4).

**Table 4:** HR of Prostate Cancer in Association with Sex, Age, Occupation, Urbanization level, Comorbidities, and Medication in Univariable and Multivariable Cox Regression Models

Variable	Crude		Adjusted^†^	
	HR	(95% CI)	HR	(95% CI)
Sleep disorder	1.51	(1.28, 1.80)***	1.42	(1.20, 1.69)***
Age, year	1.10	(1.09, 1.11)***	1.10	(1.09, 1.11)***
Occupation				
White collar	1.00	(Reference)	1.36	(1.06, 1.74)*
Blue collar	1.46	(1.22, 1.76)***	1.28	(0.99, 1.65)
Others^‡^	2.21	(1.75, 2.79)***	1.00	(Reference)
Urbanization level^†^				
1 (highest)	1.43	(1.09, 1.89)*	1.31	(0.99,1 .72)
2	1.22	(0.92, 1.61)	1.16	(0.88, 1.54)
3	1.00	(Reference)	1.00	(Reference)
4 (lowest)	1.73	(1.31, 2.29)***	1.25	(0.94, 1.66)
Comorbidity				
Hyperlipidemia	2.44	(2.05, 2.91)***	1.36	(1.13, 1.64)***
Diabetes	3.32	(2.59, 4.24)***	1.36	(1.06, 1.76)*
Hypertension	4.49	(3.77, 5.34)***	0.94	(0.75, 1.18)
Urinary stones	1.28	(0.94, 1.74)		
Urinary tract infection	2.17	(1.66, 2.83)***	1.07	(0.82, 1.40)
Obesity	1.07	(0.40, 2.87)		
Anxiety	1.07	(0.70, 1.65)		
Depression	1.05	(0.64, 1.73)		
COPD	3.54	(2.95, 4.26)***	1.03	(0.85, 1.25)
Alcohol-related illness	0.85	(0.50, 1.45)		
Medication				
Hypnotics	1.60	(1.33, 1.92)***	0.83	(0.69, 1.00)
Antihypertensives	4.29	(3.61, 5.08)***	1.17	(0.94, 1.46)
PSA screening	4.38	(3.69, 5.19)***	1.99	(1.67, 2.36)***

Crude HR, relative hazard ratio; Adjusted HR^†^: multivariable analysis with adjustment for age; occupation category; urbanization level; comorbidities of hyperlipidemia, diabetes, hypertension, urinary stones, urinary tract infection, and COPD, and medication of hypnotics as well as antihypertensive medication, and PSA screening; COPD: chronic obstructive pulmonary disease

*p < 0.05, **p < 0.01, ***p < 0.001

## Discussion

Previous studies have focused on the impact of night shift work and circadian rhythm disorders on cancer risks. [23, 24] We investigated the incidence and risk of prostate cancer in patients with SDs in an Asian population-based cohort study. Our study showed that the men with SDs displayed a greater incidence of prostate cancer than did the men without SDs (9.56 vs 6.36 per 10,000 person-y). The incidence of prostate cancer in our SD cohort was higher than that (4.79 per 10,000) in Taiwan Cancer Registry Database in 2012. [15] The possible reason may be related to considerable comorbidities and poor sleep quality in our SD cohort. [4, 5] Despite we assembled the non-SD cohort by randomly frequency-matched age, age (5-y intervals), occupation, urbanization level, comorbidities, and medications, the proportion of urinary tract infection and PSA screening were higher in the SD cohort than in the non-SD cohort. After adjustment for age, comorbidities, medication, and PSA screening, the men in the SD cohort still had a 1.42-fold increased risk of prostate cancer compared with the men in the non-SD cohort.

The incidence and risk of prostate cancer in our study were different from AGES-Reykjavik cohort study, which used questionnaires to investigate 2012 older men with sleep problems in Iceland and found that 135 of them (6.4%) were diagnosed with prostate cancer during follow-up. [12] However, Sigurdardottir et al. did not evaluate the effect of demographics, comorbidities, and hypnotic use.

The possible biological mechanism of SDs being associated with increased prostate cancer risk remains unclear. Men with reported sleep problems had lower morning levels of urinary 6-sulfatoxymelatonin, which are associated with increased risk of prostate cancer. [25] The 6-sulfatoxymelatonin in urine is the major enzymatic metabolite of melatonin. Melatonin has been observed to inhibit cancer development and growth in both in vitro and in vivo experimental models. [10, 26] Kao et al. indicated that hypnotics may relate to risk of prostate cancer. [27] We found the incidence of prostate cancer higher in patients with hypnotic use than that in patients without hypnotic use. However, the use of hypnotics was not an independent risk factor of prostate cancer in the multivariable Cox regression model.

A reciprocal interaction and regulation between sleep and the immune system exists. A lack of sleep can lead to immune suppression and activate cancer-stimulatory cytokines. [28–30] Studies have reported that patients with SDs are associated with unhealthy habits including excessive alcohol consumption and smoking, which are related to prostate cancer risk. [31–34] COPD is strongly correlated with smoking. [35] We used COPD and alcohol-related illness to evaluate smoking and alcohol consumption habits. The SD cohort exhibited significantly higher proportion of COPD and alcohol-related illness than did the non-SD cohort.

The incidence and risk of prostate cancer increased exponentially with age, which finding was consistent with previous reports. [36] Among men in white-collar employment, those with SDs exhibited a substantially higher risk of developing prostate cancer compared with those without SDs. Insomnia may be considered as a clinical marker of high job strain for white-collar workers. [37] White-collar workers experiencing occupational stress may present SDs. High job strain and stress might contribute to excess risk of prostate cancer. [38] The men who resided in the highest urbanization areas exhibited significantly increased risks of prostate cancer compared with the controls residing in the lowest urbanization areas. These epiphenomena may be associated with stress-related insomnia implicated in cancer development and progression. [16, 39, 40]

Several limitations should be considered when interpreting our results. First, the LHID does not provide detailed patient information such as smoking and alcohol consumption, which may be potential confounding factors for this study. Evidence shows that hypertension, diabetes, and COPD are associated with smoking. [41, 42] Therefore, we adjusted for hypertension, diabetes, and COPD to minimize the smoking confounder. Second, lack of time-varying approach may result in misclassification of exposure. The effect of SD on the risk of prostate cancer may be underestimated because non-SD controls may experience SD before diagnosis of prostate cancer.

The strength of the study lies in the use of a large population-based sample with a longitudinal cohort design. The diagnoses of SDs were made by physicians instead of self-reported in a questionnaire survey. Researchers using the LHID can track patients throughout a study period because the NHIA is the single payer for the NHI program in Taiwan and all beneficiaries are assigned unique identification numbers. The NHIA routinely examines the validity of the reimbursement claims data through administrative and peer-review processes.

## Conclusion

We found that patients with SDs are associated with increased prostate cancer risk, which increases with age. Therefore, appropriately managing sleep problems is a crucial healthcare concern, particularly as the number of people with SDs is increasing.

## Abbreviations

aHR: Adjusted hazard ratio
CIs: Confidence intervals
COPD: Chronic obstructive pulmonary disease
HRs: Hazard ratios
ICD-9-CM: International Classification of Diseases, Ninth Revision, Clinical Modification
IRB: Institutional review board
LHID: Longitudinal Health Insurance Database
NHI: National Health Insurance
NHIA: National Health Insurance Administration
NHIRD: National Health Insurance Research Database
PSA: Prostate specific antigen
SDs: Sleep disorders

## References

[1] Tsou MT (2013). Prevalence and risk factors for insomnia in community-dwelling elderly in northern Taiwan. J Clin Gerontol Geriatr.

[2] Baglioni C, Battagliese G, Feige B, Spiegelhalder K, Nissen C, Voderholzer U, Lombardo C, Riemann D (2011). Insomnia as a predictor of depression: a meta-analytic evaluation of longitudinal epidemiological studies. J Affect Disord.

[3] Chung WS, Lin CL, Chen YF, Chiang JY, Sung FC, Chang YJ, Kao CH (2013). Sleep disorders and increased risk of subsequent acute coronary syndrome in individuals without sleep apnea: a nationwide population-based cohort study. Sleep.

[4] Chung WS, Chen YF, Lin CL, Chang SN, Hsu WH, Kao CH (2015). Sleep disorders increase the risk of venous thromboembolism in individuals without sleep apnea: a nationwide population-based cohort study in Taiwan. Sleep Med.

[5] Chung WS, Lin HH, Cheng NC (2016). The incidence and risk of herpes zoster in patients with sleep disorders: a population-based cohort study. Medicine.

[6] Hansen J (2001). Light at night, shiftwork, and breast cancer risk. J Natl Cancer Inst.

[7] Davis S, Mirick DK (2006). Circadian disruption, shift work and the risk of cancer: a summary of the evidence and studies in Seattle. Cancer Causes Control.

[8] Turek FW, Gillette MU (2004). Melatonin, sleep, and circadian rhythms: rationale for development of specific melatonin agonists. Sleep Med.

[9] Sanchez-Barcelo EJ, Mediavilla MD, Alonso-Gonzalez C, Reiter RJ (2012). Melatonin uses in oncology: breast cancer prevention and reduction of the side effects of chemotherapy and radiation. Expert Opin Investig Drugs.

[10] Jung B, Ahmad N (2006). Melatonin in cancer management: progress and promise. Cancer Res.

[11] Gapstur SM, Diver WR, Stevens VL, Carter BD, Teras LR, Jacobs EJ (2014). Work schedule, sleep duration, insomnia, and risk of fatal prostate cancer. Am J Prev Med.

[12] Sigurdardottir LG, Valdimarsdottir UA, Mucci LA, Fall K, Rider JR, Schernhammer E, Czeisler CA, Launer L, Harris T, Stampfer MJ (2013). Sleep disruption among older men and risk of prostate cancer. Cancer epidemiology, biomarkers & prevention : a publication of the American Association for Cancer Research, cosponsored by the American Society of Preventive Oncology.

[13] Markt SC, Flynn-Evans EE, Valdimarsdottir UA, Sigurdardottir LG, Tamimi RM, Batista JL, Haneuse S, Lockley SW, Stampfer M, Wilson KM (2016). Sleep duration and disruption and prostate Cancer risk: a 23-year prospective study. Cancer epidemiology, biomarkers & prevention : a publication of the American Association for Cancer Research, cosponsored by the American Society of Preventive Oncology.

[14] Wong MC, Goggins WB, Wang HH, Fung FD, Leung C, Wong SY, Ng CF, Sung JJ (2016). Global incidence and mortality for prostate Cancer: analysis of temporal patterns and trends in 36 countries. Eur Urol.

[15] Chiang CJ, Lo WC, Yang YW, You SL, Chen CJ, Lai MS (2016). Incidence and survival of adult cancer patients in Taiwan, 2002-2012. Journal of the Formosan Medical Association = Taiwan yi zhi.

[16] Segerstrom SC, Miller GE (2004). Psychological stress and the human immune system: a meta-analytic study of 30 years of inquiry. Psychol Bull.

[17] Sharp L, Donnelly D, Hegarty A, Carsin AE, Deady S, McCluskey N, Gavin A, Comber H (2014). Risk of several cancers is higher in urban areas after adjusting for socioeconomic status. Results from a two-country population-based study of 18 common cancers. Journal of urban health : bulletin of the New York Academy of Medicine.

[18] Engeland A, Tretli S, Bjorge T (2003). Height, body mass index, and prostate cancer: a follow-up of 950000 Norwegian men. Br J Cancer.

[19] Shih CJ, Chen YT, Ou SM, Yang WC, Chen TJ, Tarng DC (2014). Urinary calculi and risk of cancer: a nationwide population-based study. Medicine.

[20] Fang HF, Miao NF, Chen CD, Sithole T, Chung MH (2015). Risk of Cancer in patients with insomnia, parasomnia, and obstructive sleep apnea: a Nationwide nested case-control study. J Cancer.

[21] Iqbal U, Nguyen PA, Syed-Abdul S, Yang HC, Huang CW, Jian WS, Hsu MH, Yen Y, Li YC (2015). Is long-term use of benzodiazepine a risk for cancer?. Medicine.

[22] NHI: National Health Insurance Database. In.: National Health Insurance Administration; 2015.

[23] Kubo T, Oyama I, Nakamura T, Kunimoto M, Kadowaki K, Otomo H, Fujino Y, Fujimoto N, Matsumoto T, Matsuda S (2011). Industry-based retrospective cohort study of the risk of prostate cancer among rotating-shift workers. Int J Urol.

[24] Parent ME, El-Zein M, Rousseau MC, Pintos J, Siemiatycki J (2012). Night work and the risk of cancer among men. Am J Epidemiol.

[25] Sigurdardottir LG, Markt SC, Rider JR, Haneuse S, Fall K, Schernhammer ES, Tamimi RM, Flynn-Evans E, Batista JL, Launer L (2015). Urinary melatonin levels, sleep disruption, and risk of prostate cancer in elderly men. Eur Urol.

[26] Cutando A, Lopez-Valverde A, Arias-Santiago S, DE Vicente J, DE Diego RG (2012). Role of melatonin in cancer treatment. Anticancer Res.

[27] Kao CH, Sun LM, Su KP, Chang SN, Sung FC, Muo CH, Liang JA (2012). Benzodiazepine use possibly increases cancer risk: a population-based retrospective cohort study in Taiwan. The Journal of clinical psychiatry.

[28] Dimitrov S, Lange T, Tieken S, Fehm HL, Born J (2004). Sleep associated regulation of T helper 1/T helper 2 cytokine balance in humans. Brain Behav Immun.

[29] Blask DE (2009). Melatonin, sleep disturbance and cancer risk. Sleep Med Rev.

[30] Besedovsky L, Lange T, Born J (2012). Sleep and immune function. Pflugers Arch.

[31] Crum RM, Storr CL, Chan YF, Ford DE (2004). Sleep disturbance and risk for alcohol-related problems. Am J Psychiatry.

[32] Phillips BA, Danner FJ (1995). Cigarette smoking and sleep disturbance. Arch Intern Med.

[33] Velicer CM, Kristal A, White E (2006). Alcohol use and the risk of prostate cancer: results from the VITAL cohort study. Nutr Cancer.

[34] Huncharek M, Haddock KS, Reid R, Kupelnick B (2010). Smoking as a risk factor for prostate cancer: a meta-analysis of 24 prospective cohort studies. Am J Public Health.

[35] Laniado-Laborin R (2009). Smoking and chronic obstructive pulmonary disease (COPD). Parallel epidemics of the 21 century. Int J Environ Res Public Health.

[36] Lin WY, Chang YH, Lin CL, Kao CH, Wu HC (2017). Erectile dysfunction and the risk of prostate cancer. Oncotarget.

[37] Metlaine A, Sauvet F, Gomez-Merino D, Elbaz M, Delafosse JY, Leger D, Chennaoui M (2017). Association between insomnia symptoms, job strain and burnout syndrome: a cross-sectional survey of 1300 financial workers. BMJ Open.

[38] Blanc-Lapierre A, Rousseau MC, Parent ME. Perceived workplace stress is associated with an increased risk of prostate Cancer before age 65. Front Oncol. 2017. 10.3389/fonc.2017.00269 PMC5693840 29181335

[39] Lutgendorf SK, Sood AK, Antoni MH (2010). Host factors and cancer progression: biobehavioral signaling pathways and interventions. J Clin Oncol.

[40] Antonova L, Aronson K, Mueller CR (2011). Stress and breast cancer: from epidemiology to molecular biology. Breast Cancer Res.

[41] Virdis A, Giannarelli C, Neves MF, Taddei S, Ghiadoni L (2010). Cigarette smoking and hypertension. Curr Pharm Des.

[42] Chang SA (2012). Smoking and type 2 diabetes mellitus. Diabetes Metab J.

